# Cerebral venous thrombosis after high-dose steroid in patient with multiple sclerosis: A case report

**DOI:** 10.1097/MD.0000000000034142

**Published:** 2023-06-23

**Authors:** Manmin Zhu, Wenxiu Cui, Wei Huang, Zheng Liu, Zucai Xu, Hao Huang

**Affiliations:** a Department of Neurology, Affiliated Hospital of Zunyi Medical University, Zunyi, China.

**Keywords:** cerebral venous thrombosis, multiple sclerosis, steroid

## Abstract

Multiple sclerosis (MS) is a central nervous system disease mainly mediated by immunity, which is one of the most common causes of neurological dysfunction in young people worldwide. In the acute phase, high-dose steroid therapy is effective. There are few reports about cerebral venous thrombosis (CVT) after high-dose steroid therapy.

**Patient concerns:**

We present a case of a 19-year-old female diagnosed with MS who developed a headache after high-dose steroid therapy was diagnosed with CVT. Headache symptoms improved after anticoagulant treatment.

**Diagnoses:**

MS comorbid CVT.

**Interventions:**

Anticoagulant therapy was added and hormone therapy was reduced.

**Outcomes:**

Clinical symptoms such as headache, limb numbness, and involuntary tremors in the right hand were improved, and the muscle strength of the right limb recovered to grade 4. The patient did not suffer from headaches after discharge and no abnormality in the computed tomography (CT) scan of the cephalic vein at the 5-months follow-up.

**Lessons:**

High-dose steroid therapy may be a risk factor for CVT in patients with MS. MS patients who develop headaches during high-dose steroid therapy should undergo further cranial CTV to rule out CVT.

## 1. Introduction

Multiple sclerosis (MS) is an autoimmune disease characterized by chronic, focal, and inflammatory demyelination in the central nervous system. Common adverse reactions after high-dose steroid therapy include electrolyte disturbance, elevated blood sugar, peptic ulcer, osteoporosis, etc. Acute hepatitis, sinus bradycardia, unilateral acute retinal necrosis, and intractable hiccups have also been reported recently in MS patients after high-dose steroid therapy. We present a case of a 19-year-old female diagnosed with MS who developed a headache after high-dose steroid therapy was diagnosed with cerebral venous thrombosis (CVT).

## 2. Case report

A 19-year-old female patient was admitted to the hospital on April 28, 2021, complaining of limb numbness for more than 2 weeks and involuntary shaking of the right hand for more than 1 week, and the disease course progressed slowly. Complaints of numbness gradually progressed from the distal end of both upper limbs and right lower limbs to the proximal end with symptoms of involuntary tremors in the right hand, weakness in the right limb, intermittent attacks lasting for several seconds to several minutes, and occasional dizziness. She had a history of pulmonary tuberculosis 3 years prior and left facial neuritis 1 year ago.

**Figure 1.**
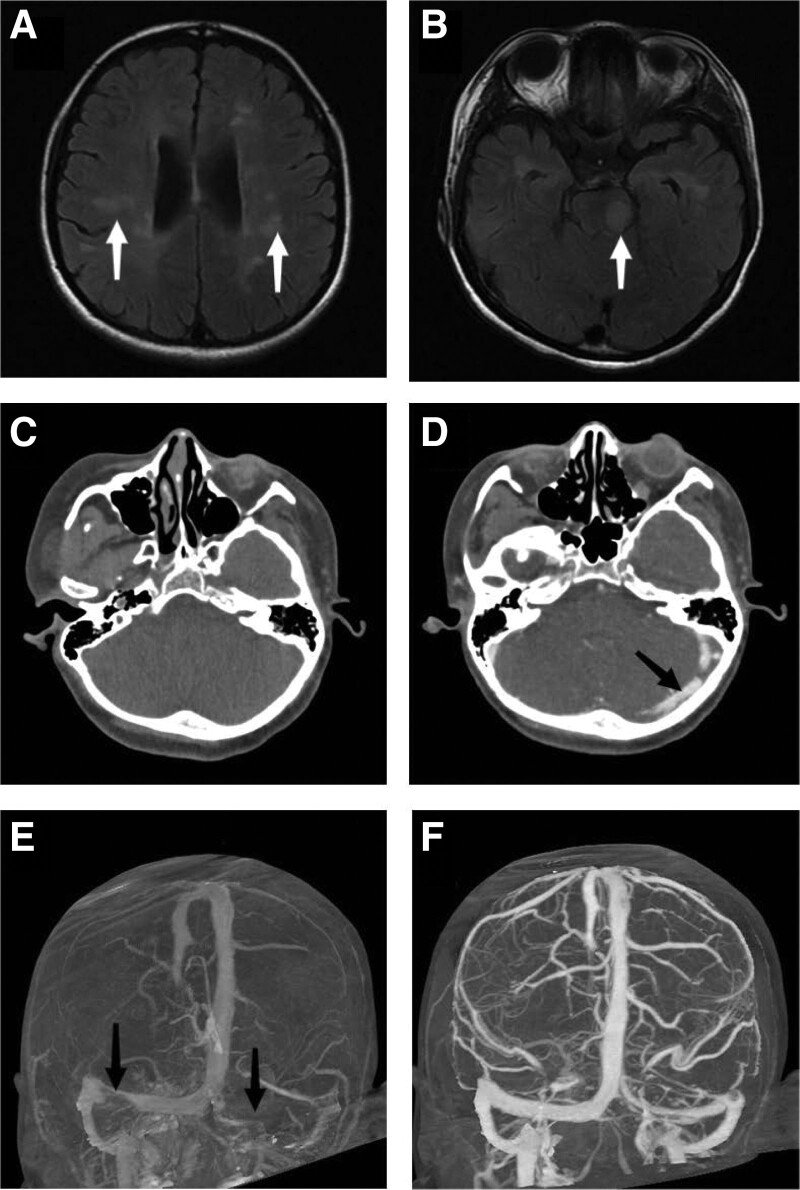
(A and B) Axial FLAIR magnetic resonance image demonstrates hyperintense. MS lesions in the pons. (C) Axial Cranial CT image without a signal change suggesting cerebral venous thrombosis on admission. (D) After 8 days of high-dose steroid treatment, cranial CT demonstrates the left sigmoid sinus, transverse sinus, and confluent sinus was hyperdense. (E) Cephalic venous CT demonstrates thrombosis in the bilateral transverse and left sigmoid sinus. (F) After anticoagulation therapy, no cerebral venous thrombosis was found on cephalic venous CT at 5-mo follow-up. CT = computed tomography, FLAIR = fluid attenuated inversion recovery, MS = multiple sclerosis.

On admission, it was suggested that the patient get her nervous system checked. The patient had a normal level of consciousness, spoke normally, limited abduction in the right and left eyes, parallel right tongue extension, weakened pharyngeal reflex, and normal functions of other cranial nerves, brainstem, bladder, and intestinal tract functions. The muscle strength of the right limb was grade 4, and that of the left limb was grade 5 with normal function. Deep tendon reflexes of both lower limbs were considered to have hyperreflexia function. A sensory examination was performed, and tingling in the right limb and left fingertip was found to be decreased; there were signs of eye closure, and the right finger nose test and calcaneal knee tibia test were positive. In addition, the bilateral Chaddock sign and right ankle clonus were positive. Head magnetic resonance imaging plain scan + enhancement: The presence of multiple nodular long T2 signals was seen in the pons, bilateral internal capsule, and bilateral frontotemporal parietal, occipital lobe, fluid attenuated inversion recovery showed high signal intensity (Fig. 1A and B). Cervical vertebrae MRI: Cervical spinal cord abnormal signal, they were considered as demyelinating and inflammatory lesions. Skull computed tomography (CT): A mild hydrocephalus nature was shown by CT scan; no cerebral CVT (Fig. 1C); routine blood examination, coagulation function, D-dimer, electrolyte, liver function, kidney function, blood glucose, blood lipid, myocardial enzyme, urine and stool tests were normal. Combined with the medical history, physical examination and auxiliary examination, she was diagnosed with MS, so we further performed lumbar puncture (LP) and electrophysiological examination.

Intracranial pressure of LP: The opening pressure was applied with 60 mmH2O to extract the cerebrospinal fluid (CSF), which appeared as a clear and transparent fluid. The total cell number was 333 × 106/L, of which the White blood cell count was 36 × 106/L. The CSF was analyzed for biochemical abnormalities. After performing the immunological assays, it was observed that the oligoclonal band (OB) of IgG in the CSF was positive for OB, however, the serum IgG OB was negative for OB.

Electrophysiological examination: The differentiation of cortical potential was stimulated by the right lower limb and had a poor response; thus, the amplitude of P40 was significantly decreased, and the latency was slightly prolonged. The cortical potential of the right upper limb stimulation was poorly differentiated, the amplitude of N20 was significantly decreased, and the latency was kept normal. Hence, the final diagnosis was MS.

On April 30, the patient began treatment with an intravenous infusion of high-dose hormone (methylprednisolone 1000 mg/day); the dose was subsequently reduced to 500 mg/day for 3 days. After 8 days of hormone treatment, she developed a headache, mainly characterized by an intermittent pinching sensation on the right posterior occipital. The left sigmoid sinus, transverse sinus, and confluent sinus was hyperdense at cranial CT (Fig. 1D). CT scan of the cephalic vein: The formation of emboli in the left sigmoid sinus, transverse sinus, straight sinus, and sinus confluence, and right transverse sinus (Fig. 1E). CT scan of the cervical vein: The filling defect of the left internal jugular vein and formation of multiple emboli was scanned, and further coagulation function was assessed. It was found that the fibrinogen level was about 1.86 g/L, plasma prothrombin time was 8.80 seconds, and the activated partial thromboplastin time was 22.10 seconds. The D-dimer was also estimated and found to be 0.58 μg/mL. Then the patient was diagnosed with CVT, and received anticoagulant therapy consisting of low-molecular-weight heparin calcium heparin. After 1 day of anticoagulant treatment, the headache subsided, but bilateral leg pain occurred. Venous color Doppler examination of the lower extremities showed an intermuscular venous thrombosis in the right leg. The anticoagulant therapy was continued, and hormone therapy was reduced rapidly. The symptoms of bilateral leg pain were significantly reduced after 4 days of prednisone treatment, and no headache or leg pain was reported after 10 days. After 17 days, CT imaging of the head vein showed left jugular vein thrombosis, the left sigmoid sinus and transverse sinus were small. After 24 days, CT imaging of the head vein was repeated and diagnosed with the same findings. After 29 days, the patient was treated with rivaroxaban tablets orally.

At the time of discharge, clinical symptoms such as headache, limb numbness, and involuntary tremors in the right hand were improved, and the muscle strength of the right limb recovered to grade 4. The patient did not suffer from headaches after discharge and no abnormality in the CT scan of the cephalic vein at the 5-months follow-up (Fig. 1F).

## 3. Conclusions

MS is an autoimmune-mediated disease of the central nervous system, often accompanied by inflammation, demyelination, and neurodegeneration, affecting approximately 2.3 million people worldwide,^[1]^ and is currently incurable. The onset of MS is usually between the ages of 20 and 40, and it is common in women. The common treatment in the acute phase is high-dose steroid pulse therapy. We present a case of a 19-year-old female diagnosed with MS who developed a headache after 8 days of high-dose steroid therapy was diagnosed with CVT. This case tells us that patients with MS who develops headaches during high-dose steroid therapy should consider the possibility of CVT.

CVT has potentially serious effects and high neurological morbidity and mortality. Risk factors include oral contraceptives, pregnancy, puerperium, LP, local and systemic infections, cancer, acquired prethrombotic conditions (e.g., hyperhomocysteinemia, nephrotic syndrome), inflammatory diseases, blood diseases, nervous system diseases (e.g., dural arteriovenous malformations, spontaneous low intracranial pressure), and drugs.

There are few reports about the mechanism of CVT during high-dose steroids in patients with MS. The possible mechanism is that the high-dose steroid might cause vascular endothelial cell injury that changes the blood flow state, an increase in the blood coagulation condition (platelet, coagulation factor increase or fibrinolysis system activity decreased), and so on. Therefore, it is easy to form a CVT due to the lack of a muscular layer of the cerebral venous sinus, inelasticity, no valve, and slow blood flow velocity.^[2]^

CVT is considered a rare complication of LP, with 16% to 19.6% of patients with CVT having a history of LP.^[3–5]^ It has been suggested that there is a decrease in CSF pressure after LP, which leads to a caudal sagging effect and damage to the fragile venous endothelial cell wall that might trigger venous vasodilation and congestion, leading to CVT. However, in almost all cases, when LP plays a role in CVT, there is 1 or more susceptible factors such as the use of high-dose steroid, inherited thrombophilia, malignancy, the postpartum state, oral contra-ceptives and reduced activity of Protein C and Protein S.^[4,6]^ Studies have shown that CVT occurs after high-dose steroid therapy in the absence of LP history and other risk factors.^[7]^ These results suggest that high-dose steroid therapy may be an independent risk factor for CVT. The proportion of CVT occurring after high-dose steroid therapy was as high as that of protein C or S deficiency.^[8]^ Prophylactic anticoagulant therapy following high-dose steroid hormone therapy may be necessary in acute episodes of MS.^[6]^ In addition to LP and high-dose steroid therapy, it has been reported that patients with MS develop CVT without LP, high-dose steroid therapy, or any other risk factor. However, there is no direct evidence confirming the direct relationship between MS and CVT in the literature.

To summarize, high-dose steroid therapy may be a risk factor for CVT in patients with MS. MS patients who develop headaches during high-dose steroid therapy should undergo further cranial CTV to rule out CVT.

## Author contributions

**Data curation:** Wei Huang, Zheng Liu.

**Formal analysis:** Wei Huang, Zheng Liu.

**Supervision:** Zucai Xu, Hao Huang.

**Validation:** Hao Huang.

**Writing – original draft:** Manmin Zhu, Wenxiu Cui.

**Writing – review & editing:** Manmin Zhu.
